# Effectiveness and Cost-effectiveness of Online Brief Mindfulness-based Cognitive Therapy for the Improvement of Productivity in the Workplace: Study Protocol for a Randomized Controlled Trial

**DOI:** 10.2196/36012

**Published:** 2022-06-13

**Authors:** Mitsuhiro Sado, Masashi Yamada, Akira Ninomiya, Maki Nagaoka, Naho Goto, Akihiro Koreki, Atsuo Nakagawa, Zindel Segal, Masaru Mimura

**Affiliations:** 1 Department of Neuropsychiatry Keio University School of Medicine Tokyo Japan; 2 Center for Stress Research Keio University Tokyo Japan; 3 Department of Psychiatry Shimofusa Psychiatric Medical Center National Hospital Organization Chiba Japan; 4 Graduate Department Psychological Clinical Science University of Toronto Scarborough Toronto, ON Canada

**Keywords:** mindfulness-based cognitive therapy, mindfulness, cognitive therapy, occupational health, workplace, randomized controlled trial, cost-effectiveness, cost, online, internet-based, eHealth, mental health, heath outcome, work, stress, burnout, productivity, employee

## Abstract

**Background:**

Numerous studies have demonstrated the effectiveness of mindfulness-based programs (MBPs) among both clinical and nonclinical populations. These data document positive impacts in the workplace, including reducing perceived stress and burnout and increasing well-being. However, the effectiveness for productivity, which is of most interest to managers and administrators, is still unclear. In addition, MBPs in the workplace tend to be modified by reducing the number of the program sessions or delivering content online to improve accessibility. To date, however, the impact of MBPs that feature these modifications on productivity in the workplace has not been investigated.

**Objective:**

The study aims to investigate the effectiveness and cost-effectiveness of online-delivered brief mindfulness-based cognitive therapy (bMBCT) for improving productivity and other work-related outcomes among healthy workers compared to the waitlist control.

**Methods:**

We will conduct a 4-week randomized controlled trial (RCT) with a 6-month follow-up. Employees are included in the study if they (1) are between the ages of 20 and 65 years and (2) work longer than 30 hours weekly. Employees are randomly allocated to either the bMBCT group or the waitlist control group. The primary outcome of the study is the mean difference of productivity measured by the World Health Organization Health and Work Performance Questionnaire (WHO-HPQ) between the groups at 4, 16, and 28 weeks. Secondary outcomes include several clinical outcomes and health economics evaluation.

**Results:**

We started recruiting participants in August 2021, and the intervention began in October 2021. A total of 104 participants have been enrolled in the study as of October 2021. The intervention is scheduled to be completed in December 2023. Data collection will be completed by the end of January 2024.

**Conclusions:**

The novelty of the study is that (1) it will investigate bMBCT’s effectiveness on productivity, which is still unclear, and (2) samples are recruited from 3 companies in different industries. The limitations of the study are that (1) all measures assessed are in self-report format and (2) we lack an active control group. This study has the potential to provide new data on the relationship between MBPs and occupational health and productivity.

**Trial Registration:**

University Hospital Medical Information Network Clinical Trials Registry UMIN000044721; https://tinyurl.com/4e2fh873

**International Registered Report Identifier (IRRID):**

DERR1-10.2196/36012

## Introduction

### Background

Mindfulness-based programs (MBPs) are defined as interventions featuring systematic and sustained training in formal and informal mindfulness meditation practices; this training is recognized as central to both the therapeutic approach and the underpinning theoretical model [1]. Numerous clinical trials have demonstrated the effectiveness of MBPs in clinical populations, including patients with depression [2-4], anxiety [5-11], cancer [12-15], and pain [16-20]. These results have been affirmed by the findings of several meta-analyses [21-25].

However, it should be noted that the effectiveness of MBPs is not restricted to clinical populations. MBPs have been shown to be effective in nonclinical populations as well. Existing evidence indicates that MBPs are efficacious for the improvement of stress, sleep, quality of life, and subjective well-being in healthy individuals [26-38]. This is also applicable in the context of occupational health. In their latest meta-analysis of 23 randomized controlled trials (RCTs), Bartlett et al [39] endorsed the effectiveness of MBPs in the workplace for the improvement of numerous clinical factors, such as perceived stress, psychological distress, depression, anxiety, burnout, well-being, and sleep.

### Rationale for the Study

Regarding productivity, which is of most interest to managers, Bartlett et al [39] concluded that productivity was assessed too inconsistently and infrequently for related results to be included in their meta-analysis; thus, they reported the results narratively. Three previous studies have indicated that absenteeism and presenteeism post-MBP intervention show a positive but nonsignificant tendency [35,40,41]; however, one study indicated no effect in that regard [42,43]. Regarding work engagement, although 1 study showed null results [42,43], another study revealed a significant positive effect of MBPs on work engagement [44]. As the inconsistency of the results of previous studies indicates, the effect of MBPs on productivity in the workplace is still unclear. In addition, certain aspects of MBPs, including the delivery mode (eg, online delivery) and the number of sessions, tend to be modified when applied in the workplace to improve accessibility. These modifications are likely to be applied more frequently because of the effects of the COVID-19 pandemic. However, in 85% of the studies included in the meta-analysis by Bartlett et al [39], the interventions were delivered face-to-face and the average number of sessions offered was not necessarily small (7). Thus, the effectiveness of MBPs delivered online and in a small number of sessions for improved accessibility has not been sufficiently investigated, especially in terms of productivity in the workplace.

Therefore, we developed a brief mindfulness-based cognitive therapy (bMBCT) program, which consists of four 1.5-hour sessions, to evaluate the effectiveness and cost-effectiveness of online bMBCT for the improvement of productivity and other work-related indicators compared to the waitlist control.

### Aim

The aim of this study is to investigate the effectiveness and cost-effectiveness of online bMBCT for the improvement of productivity and other work-related outcomes among healthy workers compared to the waitlist control.

## Methods

### Participants

We started recruiting participants in August 2021. The intervention is ongoing and is scheduled to be completed in December 2023. The study is being conducted at the Keio University Center for Stress Research in Tokyo, Japan. The participants are being recruited from among the employees of 3 companies in different industries: Daiwa Securities Group Inc (security), Kumon Institute of Education Co, Ltd (education), and Nichirei Corporation (processed foods). Participants are eligible for the study if they meet the following criteria: (1) aged between 20 and 65 years, (2) work longer than 30 hours weekly, (3) have no history of sick leaves longer than 1 month due to mental disorders or have recovered for longer than 6 months after a sick leave, (4) have physical illnesses but are judged fit to participate in the research by the investigators, (5) score 8 or less in the absolute presenteeism item of the World Health Organization Health and Work Performance Questionnaire (WHO-HPQ), (6) can participate in the intervention and respond to the questionnaires via the internet, and (7) can provide written informed consent. Eligible participants are excluded if they (1) have previously participated in a mindfulness-based intervention for 8 weeks or longer, (2) are unlikely to participate during the research period (eg, they plan to move/relocate), and (3) are judged by the investigators as unfit to participate in the intervention due to physical conditions and other reasons (eg, unstable internet connection). Candidates who score higher than 8 on the WHO-HPQ absolute presenteeism item are allowed to participate in the intervention but will not be included in the RCT. The obtained data will be included in the multivariable analysis described later.

### Enrollment

Prospective participants, who applied for the study via recruiting announcements delivered at each company, received a link to the web screening from the research cooperator at each company. If they passed the web screening, an announcement of the video group orientation was made. In the online group orientation, the research investigators provided written and oral explanations of the study, which included full descriptions of the purpose, significance, and methods of the study; the risks and benefits of participation; and the requirements for consent. Prospective participants were also offered an opportunity to ask questions. The investigators recorded the method, content, and date of the explanations provided. After the online group orientation, individual video interviews were held and the research investigator evaluated whether the participants met the inclusion criteria. Since obtaining written informed consent via online sessions was not feasible, all included participants provided informed consent verbally. The records were converted into PDF files, saved in an electronic storage medium, and stored in a lockable cabinet at the Stress Research Center.

### Baseline Assessment

The included participants completed the questionnaires administered for the collection of demographic and psychosocial data. The psychological assessment tools to be utilized include the WHO-HPQ, the Five Facet Mindfulness Questionnaire (FFMQ), the 9-item Utrecht Work Engagement Scale (UWES-9), the Satisfaction With Life Scale (SWLS), the Flourishing Scale (FS), the Scale of Positive and Negative Experience (SPANE), the Experiences Questionnaire (EQ), the Perceived Stress Scale (PSS), the Investigating Choice Experiments Capability Measure for Adults (ICECAP-A), Team Psychological Safety (TPS), and the Credibility/Expectancy Questionnaire (CEQ). The details of each scale are presented later in the Instruments section.

### Randomization

**Figure 1:**
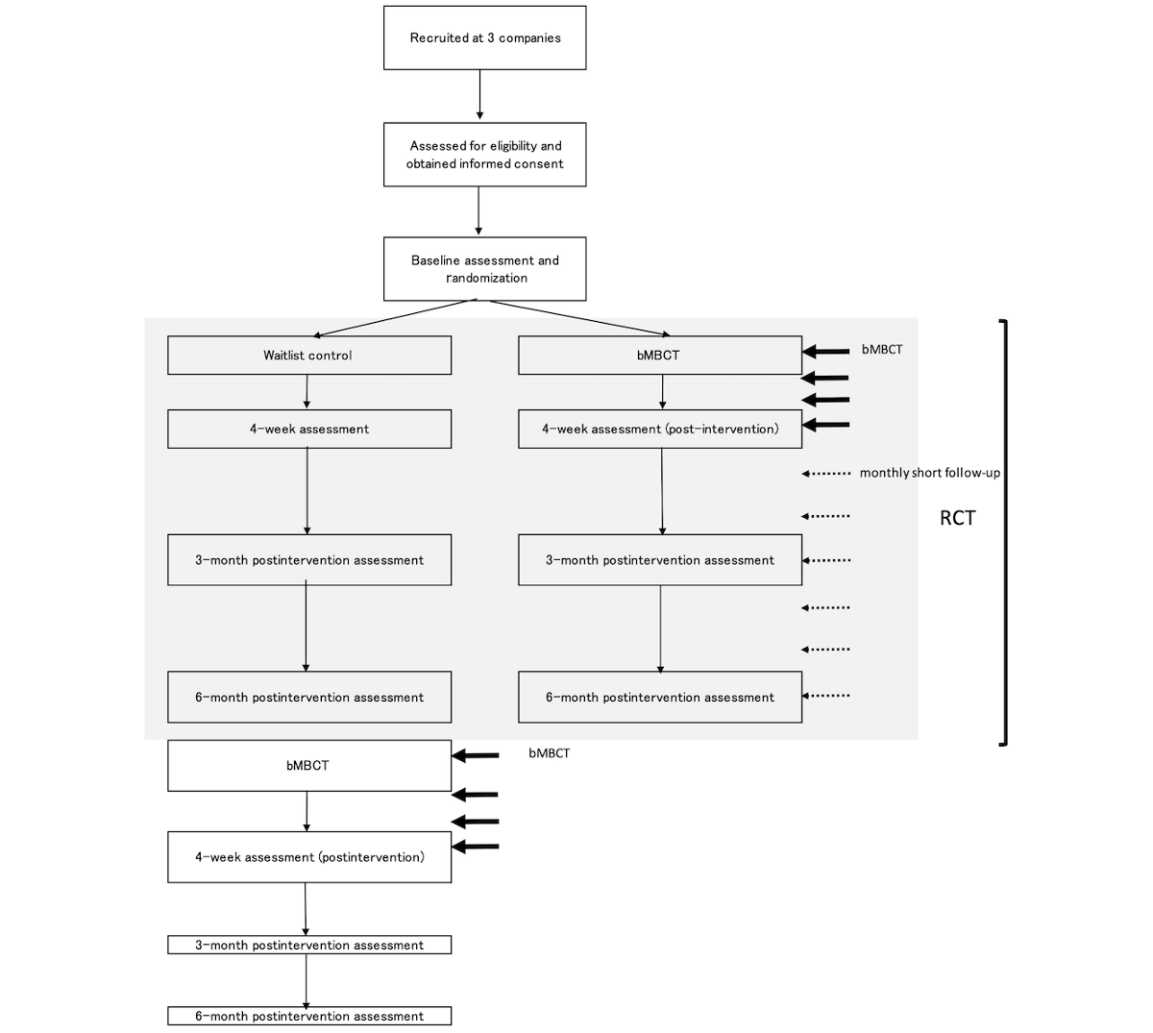
Flowchart of the study. bMBCT consists of four 1.5-hour-long sessions (only session 1 lasts for 2 hours). The follow-up session lasts for 1 hour and consists of a short meditation, experience sharing, and a question-and-answer session. bMBCT: brief version of mindfulness-based cognitive therapy; RCT: randomized controlled trial.

Eligible participants were randomly allocated to either the bMBCT or the waitlist control group (in a 1:1 ratio). The participants were assigned a computer-generated random number stratified according to company and the baseline WHO-HPQ absolute presenteeism score. The Project Management Office at the Keio Center of Clinical Research, which is an institution independent from the study group, managed the randomization process. The flow of participant recruitment is shown in Figure 1.

### Blinding

Due to the nature of psychological interventions, both the participants and the therapists were not blinded to randomization statuses. Since all measurements obtained during the study are self-reported, there were no assessors for the evaluation of the statuses of participants.

### Interventions

#### Brief Mindfulness-Based Cognitive Therapy

The included participants were offered a bMBCT program, which is a modified version of the original MBCT developed by Segal et al [45]. The modifications followed Crane et al’s [1] specifications of “warp” (essential ingredients) and “weft” (flexible ingredients) of MBPs. To retain the essential ingredients of MBPs, we included practicing all mindful meditations except mindful movement and walking meditation (provided as homework via a delivery instruction movie). In contrast, considering the difference in the delivery setting (clinical setting vs workplace) and the target population (ie, patients with depression vs healthy individuals), some modifications were made. The first is the structure of the program. With the aim of improving accessibility to the program in the workplace, the duration of each session was shortened (from 2 hours to 1.5 hours), except session 1, and the total number of sessions was reduced (from 8 sessions to 4 sessions). To reflect the difference in the target population, the lecture relevant to depression was deleted, and activity records (ie, pleasant, unpleasant, appreciation events, and nourishing and depriving activities) were introduced to enhance the improvement of participants’ well-being. The specific program contents are listed in Table 1. In the program, participants learned both cognitive approaches and mindfulness practices (eg, raisin exercise, body scan, sitting meditation, exploring difficulty, and 3-step breathing space). The participants were asked to practice meditation daily for approximately 20 minutes by listening to the pre-recorded meditation guide audios and record some activities as homework (theme depends on the session). Monthly follow-up sessions (lasts for 1 hour) will be provided to the participants for 6 months. No regular homework will be assigned during the follow-up period. During the follow-up period, the participants can access the mindfulness website developed by the research team from a smartphone or a personal computer (PC) and easily stream/download the meditation instructions.

In addition, the participants were encouraged to send their mindfulness experiences in daily life to the research team. The research team posted and shared them on the website. The research team also posted relevant articles to support participants in continuing the therapy. Participants were encouraged to meditate depending on their needs. In addition to encouraging individual participants to continue meditation, we encouraged them to develop voluntary groups to regularly meditate together outside the program. The bMBCT sessions and follow-up sessions are delivered live outside working hours via a videoconference platform.

The first, third, and fourth authors lead the sessions. The first author is a qualified mindfulness-based stress reduction teacher at the University of Massachusetts, with 12 years of experience in mindfulness practice. The other 2 authors have been practicing mindfulness for more than 5 years and have administered MBCTs 5 times under the supervision of the first author.

**Table 1:** Themes and contents of the program.

Session	Theme	Content
1	Doing mode and being mode/wondering mind	Meditation: raisin exercise/body scan Exercise: What is mindfulness? Homework: mindfulness in a daily activity/body scan.
2	Sensation continues to change/thoughts are not fact	Meditation: mindful movement/breath and body meditation/3-step breathing space Exercise: scenario exercise (thought and feeling exercise) Homework: body scan or breath and body meditation/mindful movement/pleasant-event calendar/3-step breathing space
3	Thoughts are a phenomenon in the mind/thought-body-behavior-mood are connected	Meditation: sound and thought meditation/3-step breathing space Exercise: nourishing activity Homework: sound and thought meditation/unpleasant-event calendar/nourishing activity/3-step breathing space
4	Exploring difficulty	Meditation: exploring difficulty/3-step breathing space Exercise: reflection of the course (“Why am I here?” exercise) Homework: no regular homework (encouraged to practice depending on their need)

### Control Group

Participants in the control group will wait until the intervention group has completed the intervention. During this waiting period, they have been requested not to attend other mindfulness or meditation activities. After the waiting period is completed (ie, 7 months after allocation), the participants in the control group will be offered the same bMBCT but without monthly follow-up sessions.

### Outcomes

#### Primary Outcome

The primary outcome of this study is the mean difference in absolute presenteeism (measured using the WHO-HPQ) between the intervention and the waitlist control (ie, before bMBCT offered) groups at 4 weeks, 3 months, and 6 months postintervention.

#### Secondary Outcomes

The secondary outcomes are the mean differences in the FFMQ, UWES-9, SWLS, FS, SPANE, EQ, PSS, ICECAP-A, TPS, and SEC scores at 4 weeks, 3 months, and 6 months postintervention.

### Evaluation of Health Economics

Cost-effectiveness was measured by calculating the incremental cost-effectiveness ratio, which is the incremental cost divided by the incremental effectiveness between the groups. Incremental effectiveness was evaluated using quality-adjusted life-years, calculated from the weighted ICECAP-A scores. The analyses were conducted from a company’s perspective (ie, direct cost). Cost benefit will be evaluated using the net monetary benefit, which is calculated by subtracting the incremental cost needed for the intervention from the net monetary benefit of incremental productivity, weighted by the WHO-HPQ score.

#### Multivariable Analysis

To investigate factors that predict or mediate clinical outcomes, multivariate analysis will be conducted using factors obtained during the study. The details of the analytical methodology will be presented separately.

### Instruments

#### World Health Organization Health and Work Performance Questionnaire

The WHO-HPQ is a self-report instrument designed to estimate the workplace costs of health problems in terms of self-reported sickness absences and reduced job performance (presenteeism). Presenteeism is assessed using the following questions: “On a scale of 0-10, where 0 is the worst job performance anyone could have at your job and 10 is the performance of a top worker, how would you rate the usual performance of most workers in a job similar to yours?” and “Using the same 0-10 scale, how would you rate your overall job performance on the days you worked during the past 4 weeks?” A low presenteeism score indicates poor performance [46].

#### Five Facet Mindfulness Questionnaire

This tool is a self-report questionnaire used to assess dispositional mindfulness. It includes 5 factors, which are extracted based on a factor analysis of 5 mindfulness questionnaires developed independently. The 5 facets are observing, describing, acting with awareness, not judging one’s inner experience, and not reacting to one’s inner experience. Total scores range from 39 to 195. Higher scores indicate greater levels of dispositional mindfulness [47].

#### The 9-Item Utrecht Work Engagement Scale

The UWES-9 is a 9-item self-report questionnaire that is widely used to measure work engagement across countries. It is hypothesized to assess 3 aspects of work engagement: vigor, dedication, and absorption. Each aspect includes 3 items. The scores range from 0 to 54. Higher scores indicate higher work engagement [48].

#### Satisfaction With Life Scale

This is a 5-item self-report questionnaire used to evaluate the cognitive aspects of subjective well-being. Scores for each subscale range from 1 (*strongly disagree*) to 7 (*strongly agree*). Total scores range from 5 to 35, with higher scores indicating higher satisfaction [49].

#### Flourishing Scale

This scale includes 8 items relevant to significant aspects of human functioning, ranging from positive relationships to feelings of competence, meaning, and purpose in life. Responses to each item are rated on a scale of 1-7, ranging from *strong disagreement* to *strong agreement*. Possible total scores range from 8 (*strong disagreement* with all items) to 56 (*strong agreement* with all items). High scores indicate that respondents view themselves positively in important areas of functioning [50].

#### Scale of Positive and Negative Experience

This measure is a 12-item scale that assesses positive experiences (6 items) and negative experiences (6 items). Owing to the generality of the items included in this scale, it can assess pleasant and unpleasant feelings that are the focus of most scales and can also reflect other conditions, such as interest, flow, positive engagement, and physical pleasure. Positive (SPANE-P) and negative (SPANE-N) scale scores range from 6 to 30. Higher scores indicate a higher positive or negative affective status. Subtraction of the negative score from the positive score yields the SPANE-B score, which is between −24 and 24 [50].

#### Experiences Questionnaire

The EQ is a 20-item self-report measure based on a 5-point Likert scale that ranges from 1 (*never*) to 5 (*always*). The total score ranges from 20 to 100. The scale focuses on decentering, defined as the ability to view the self as separate and different from its own thoughts, the capacity for nonreacting to negative experiences, and the ability to be self-compassionate. The EQ has been found to be reliable, and convergent and discriminant validities have been established for both general and clinical samples. The EQ is also internally consistent, with temporal stability over a 1-month period and good convergent validity [51,52].

#### Perceived Stress Scale

The PSS was developed to assess the degree to which situations in one’s life are appraised as stressful. The scale has 2 versions: the 14-item version (PSS-14) and the 10-item version (PSS-10), which is similar to the 14-item version but with 4 items removed. We used the PSS-10 in this study. This scale is used to assess perceived stressful experiences or stress responses in the previous month. Each item is rated on a 5-point Likert scale ranging from 4 (*never*) to 0 (*very often*) to identify positive experiences or responses. Total scores range from 0 to 40. Higher scores indicate higher stress levels [53].

#### Investigating Choice Experiments Capability Measure for Adults

ICECAP-A was developed to measure capability well-being in adults, which the existing health-related quality-of-life scales have not been able to adequately capture. It is a scale of 5 attributes, each with 4 levels. It provides a single index value for well-being utility, either 0 or 1. A higher score indicates better well-being status [54].

#### Team Psychological Safety

The TPS is a scale developed for the assessment of a shared belief held by members of a team that the team is safe for interpersonal risk taking. This is a 8-item tool with a 7-point Likert scale. A higher score indicates that the respondent feels better psychological safety in the team [55].

#### Credibility/Expectancy Questionnaire

The CEQ is a quick and easy-to-administer scale used to measure treatment expectancy and rationale credibility in clinical trials. It consists of 6 items rated on a 9-point Likert scale, with 1 being *not at all* and 9 indicating *very logical/useful/confident/much*. Total scores range from 9 to 54. Higher scores represent higher credibility and expectancy for treatment. This scale is derived from 2 predicted factors: cognitive credibility and relatively more affective expectancy. These 2 factors are confirmed to be stable across different populations [56].

### Homework Engagement/Qualitative Data

Daily formal meditation time and the answers to open-ended questions are collected at the end of each session. The questions include the following: (1) What did you notice in this session? (2) Did you experience any difficulties in this session? (3) Do you have any comments to improve the sessions?

The validity and reliability of the original versions of all these scales have been confirmed [46-56]. Regarding the Japanese versions of the scales, the validity and reliability of all scales and questionnaires, except ICECAP, the TPS, and the CEQ, have been confirmed [57-62]. For ICECAP-A, the Japanese version of the ICECAP officially accepted by the University of Birmingham was used [63]. We adopted the Japanese version of the CEQ, which was translated by Ito et al [64] through a rigorous back-translation procedure with the permission and support of the original developer of the questionnaire. Regarding the TPS, we used the TPS questionnaire cited in the Japanese edition of *The Fearless Organization: Creating Psychological Safety in the Workplace for Learning, Innovation, and Growth* by Edmondson [65].

### Schedule for Assessments

In addition to the baseline assessment, we requested that the participants respond to these self-report assessments at 4 weeks, 3 months, and 6 months postintervention. A range of ±2 weeks from the scheduled dates for the baseline and postintervention assessments and ±4 weeks for the 3- and 6-month postintervention assessments were allowed. All assessment data were collected using the electronic patient-reported outcomes (ePRO) system. The assessment schedules are presented in Tables 2 and 3.

**Table 2:** Assessment schedule for all participants.

Process/assessment	Screening period	Intervention period					Follow-up period						
		Week 1	Week 2	Week 3	Week 4	1 month		2 months	3 months	4 months	5 months	6 months	
Screening	A^a^	N/A^b^	N/A	N/A	N/A	N/A		N/A	N/A	N/A	N/A	N/A	
Informed consent	A	N/A	N/A	N/A	N/A	N/A		N/A	N/A	N/A	N/A	N/A	
bMBCT^c^	N/A	I^d^	I	I	I	F^e^		F	F	F	F	F	
Waitlist	N/A	N/A	N/A	N/A	N/A	N/A		N/A	N/A	N/A	N/A	N/A	
WHO-HPQ^f^	A	N/A	N/A	N/A	A	N/A		N/A	A	N/A	N/A	A	
FFMQ^g^	A	N/A	N/A	N/A	A	N/A		N/A	A	N/A	N/A	A	
UWES-9^h^	A	N/A	N/A	N/A	A	N/A		N/A	A	N/A	N/A	A	
SWLS^i^	A	N/A	N/A	N/A	A	N/A		N/A	A	N/A	N/A	A	
FS^j^	A	N/A	N/A	N/A	A	N/A		N/A	A	N/A	N/A	A	
SPANE^k^	A	N/A	N/A	N/A	A	N/A		N/A	A	N/A	N/A	A	
EQ^l^	A	N/A	N/A	N/A	A	N/A		N/A	A	N/A	N/A	A	
PSS^m^	A	N/A	N/A	N/A	A	N/A		N/A	A	N/A	N/A	A	
ICECAP-A^n^	A	N/A	N/A	N/A	A	N/A		N/A	A	N/A	N/A	A	
TPS^o^	A	N/A	N/A	N/A	A	N/A		N/A	A	N/A	N/A	A	
CEQ^p^	A	N/A	N/A	N/A	A	N/A		N/A	A	N/A	N/A	A	
Health service use	A	N/A	N/A	N/A	A	N/A		N/A	A	N/A	N/A	A	
Homework engagement/qualitative data	N/A	A	A	A	A	A		A	A	A	A	A	

^a^A: assessment.

^b^N/A: not applicable.

^c^bMBCT: brief mindfulness-based cognitive therapy.

^d^I: intervention.

^e^F: follow-up.

^f^WHO-HPQ: World Health Organization Health and Work Performance Questionnaire.

^g^FFMQ: Five Facet Mindfulness Questionnaire.

^h^UWES-9: 9-item Utrecht Work Engagement Scale.

^i^SWLS: Satisfaction With Life Scale.

^j^FS: Flourishing Scale.

^k^SPANE: Scale of Positive and Negative Experience.

^l^EQ: Experiences Questionnaire.

^m^PSS: Perceived Stress Scale.

^n^ICECAP-A: Investigating Choice Experiments Capability Measure for Adults.

^o^TPS: Team Psychological Safety.

^p^CEQ: Credibility/Expectancy Questionnaire.

**Table 3:** Assessment schedule for participants in the waitlist control group, followed by a waiting period.

Process/assessment	Intervention period (followed by a waiting period)					Follow-up period (followed by a waiting period)					
	Week 1	Week 2	Week 3	Week 4	1 month		2 months	3 months	4 months	5 months	6 months
Screening	N/A^a^	N/A	N/A	N/A	N/A		N/A	N/A	N/A	N/A	N/A
Informed consent	N/A	N/A	N/A	N/A	N/A		N/A	N/A	N/A	N/A	N/A
bMBCT^b^	N/A	N/A	N/A	N/A	N/A		N/A	N/A	N/A	N/A	N/A
Waitlist	I^c^	I	I	I	N/A		N/A	N/A	N/A	N/A	N/A
WHO-HPQ^d^	N/A	N/A	N/A	A^e^	N/A		N/A	A	N/A	N/A	A
FFMQ^f^	N/A	N/A	N/A	A	N/A		N/A	A	N/A	N/A	A
UWES-9^g^	N/A	N/A	N/A	A	N/A		N/A	A	N/A	N/A	A
SWLS^h^	N/A	N/A	N/A	A	N/A		N/A	A	N/A	N/A	A
FS^i^	N/A	N/A	N/A	A	N/A		N/A	A	N/A	N/A	A
SPANE^j^	N/A	N/A	N/A	A	N/A		N/A	A	N/A	N/A	A
EQ^k^	N/A	N/A	N/A	A	N/A		N/A	A	N/A	N/A	A
PSS^l^	N/A	N/A	N/A	A	N/A		N/A	A	N/A	N/A	A
ICECAP-A^m^	N/A	N/A	N/A	A	N/A		N/A	A	N/A	N/A	A
TPS^n^	N/A	N/A	N/A	A	N/A		N/A	A	N/A	N/A	A
CEQ^o^	N/A	N/A	N/A	A	N/A		N/A	A	N/A	N/A	A
Health service use	N/A	N/A	N/A	A	N/A		N/A	A	N/A	N/A	A
Homework engagement	A	A	A	A	A		A	A	A	A	A

^a^N/A: not applicable.

^b^bMBCT: brief mindfulness-based cognitive therapy.

^c^I: intervention.

^d^WHO-HPQ: World Health Organization Health and Work Performance Questionnaire.

^e^A: assessment.

^f^FFMQ: Five Facet Mindfulness Questionnaire.

^g^UWES-9: 9-item Utrecht Work Engagement Scale.

^h^SWLS: Satisfaction With Life Scale.

^i^FS: Flourishing Scale.

^j^SPANE: Scale of Positive and Negative Experience.

^k^EQ: Experiences Questionnaire.

^l^PSS: Perceived Stress Scale.

^m^ICECAP-A: Investigating Choice Experiments Capability Measure for Adults.

^n^TPS: Team Psychological Safety.

^o^CEQ: Credibility/Expectancy Questionnaire.

### Sample Size

The primary outcome will be analyzed using the mixed model repeated measurement (MMRM) method to compare the amount of change between groups before and after the intervention (α .05, β .10). To the best of our knowledge, no previous studies have indicated the effect size of MBPs on presenteeism among employees. The sample size was calculated based on the results of a study that showed the effect of MBPs on the quality of life (effect size=0.44) [37]. We estimated that a total of 166 participants are required; however, the sample size was set at a maximum of 220 participants, considering the dropout rate (assumed to be 25%). The dropout rate was referred to the study of an online MBP in the workplace by Aikens et al [44], which reported the dropout rate was 23%.

### Statistical Analysis

Statistical analyses and reporting of this trial will be based on the intention-to-treat approach. Analyses with complete samples will be also performed to verify the robustness of the results.

The full analysis data set will include all randomized subjects who underwent at least 1 procedure of the intervention.

For baseline variables, we will generate summary statistics with proportions and frequencies for categorical variables and means and SDs for continuous data. For primary and secondary outcome analyses, we will analyze the mean changes from baseline using the MMRM method. Analyses conducted using the MMRM method will include the fixed and categorical effects of intervention, time, and the intervention × time interaction. Imputation will not be performed for missing values, because mixed models can deal with missing data through the maximum likelihood. All comparisons are planned, and all *P* values will be 2-sided. The significance level will be set at 5% for all statistical analyses. All analyses will be conducted using Stata version 16 (StataCorp).

We will also conduct a multivariate analysis to verify the predictors and mediators of the primary and secondary outcomes. A detailed analytical plan will be presented separately.

### Adverse Events

The research team will immediately contact the Ethics Review Committee at the Keio University School of Medicine if the participants report any serious adverse events. Serious adverse events are defined as follows: (1) death by suicide, (2) death by nonsuicide, (3) a suicide attempt (self-injurious behavior that admits a suicide attempt), (4) an event that may lead to death, (5) psychiatric hospitalization, (6) general hospitalization due to an adverse event, (7) disability leading to inactivity due to an adverse event, or (8) any events judged to be medically serious based on the Japanese version of Common Terminology Criteria for Adverse Events v3.0 by the Japan Clinical Oncology Group/the Japan Society of Clinical Oncology [66]. Participants are asked to report any adverse events at the end of each session.

### Ethics Approval

The authors confirmed that all procedures complied with the ethical standards of the relevant national and institutional committees on human experimentation and with the tenets of the 1975 Declaration of Helsinki, revised in 2008. All procedures involving human participants and patients were approved by the Ethics Review Committee of the Keio University School of Medicine (Reference 2021-0101). All included participants provided informed consent after all procedures were explained in detail. They were allowed to withdraw their consent at any time without any negative consequences.

### Dissemination

We expect the results of our research to be presented at conferences and published as papers in academic journals. The results of this research will adhere to the Consolidated Standards of Reporting Trials (CONSORT) statement.

## Results

We started recruiting participants in August 2021, and the intervention began in October 2021. A total of 104 participants have been enrolled in the study as of October 2021. The intervention is scheduled to be completed in December 2023. Data collection will be completed by the end of January 2024.

## Discussion

### Summary

The aim of this study is to evaluate the effectiveness and cost-effectiveness of bMBCT in the workplace, especially for productivity. This study is valuable because it will fill certain gaps in the existing MBCT research. First, previous studies have revealed that the effectiveness of MBPs for physical and psychological indicators (eg, stress, burnout, and sleep well-being,) is promising; however, its effects on productivity are still unclear. This study is expected to clarify this important aspect of the use of MBPs in the workplace. Second, as the participants of this study are being recruited from 3 companies in different industries (security, education, and processed foods), the generalizability of the study findings will be enhanced because the cohorts of previous studies were recruited from a single company. Third, this study will focus on the evaluation of the effectiveness of online bMBCT in the workplace. Since the accumulated evidence on the effect of online bMBCT is still insufficient, the results of this study will advance the understanding of the use and effect of online bMBCT in the workplace. This is particularly important in the current scenario where many employees are forced to work from home. Furthermore, we will compare the effectiveness of bMBCT with follow-up and bMBCT without follow-up. The participants in the waitlist control group will receive bMBCT without follow-up once the waiting period is completed. Although this is not a direct RCT-based comparison, we can preliminarily evaluate the differences between the effect of bMBCT with and bMBCT without follow-up. Finally, we included a scale for the assessment of an organization’s culture. Although previous studies have revealed that an individual’s productivity is affected by individual factors and their organization’s culture, such as team psychological safety, the interaction between individual and organizational factors has not been assessed to date. In the multivariate analysis, we plan to investigate the effect of differences in an organization’s culture on the study outcomes. We are aware that focusing on productivity may possibly induce the increase of the “craving mind” of the participants, which is contrary to the purpose of MBPs. Such misuse of MBPs could have a negative impact on employees’ health and well-being. Therefore, we will also assess these outcomes as well as productivity in order to evaluate the suitability of our MBP.

### Limitations

This study has the following limitations. First, as all measures assessed are in self-report format, uncertainty regarding objectivity remains. Second, since we do not have an active control group (we set the waitlist as the control group), we cannot detect the effects that are specifically attributable to bMBCT. However, considering that the main objective of this study is to investigate the effectiveness of augmenting typical daily life with bMBCT rather than to assess the efficacy of bMBCT, we considered the research design to be acceptable for that purpose. Third, although we encourage the participants to report any serious adverse events at the end of each session, we will not assess mild-to-moderate adverse events. Therefore, the adverse events caused by this intervention might be underestimated. Despite the aforementioned limitations, we believe that this study will provide valuable information for future clinical trials in this field.

### Conclusion

This study has the potential to provide new data on the relationship between MBPs and occupational health and productivity.

## Abbreviations

bMBCT: brief mindfulness-based cognitive therapy
CEQ: Credibility/Expectancy Questionnaire
EQ: Experiences Questionnaire
FFMQ: Five Facet Mindfulness Questionnaire
FS: Flourishing Scale
ICECAP-A: Investigating Choice Experiments Capability Measure for Adults
MBP: mindfulness-based program
MMRM: mixed model repeated measurement
PSS: Perceived Stress Scale
RCT: randomized controlled trial
SPANE: Scale of Positive and Negative Experience
SWLS: Satisfaction With Life Scale
TPS: Team Psychological Safety
UWEC-9: 9-item Utrecht Work Engagement Scale
WHO-HPQ: World Health Organization Heath and Work Performance Questionnaire

## Data Availability

The data sets are available from the corresponding author on reasonable request.
